# Association of Glycoprotein IIIa PlA1/A2 Polymorphism with Risk of Stroke: Updated Meta-Analysis

**DOI:** 10.3390/cimb46060321

**Published:** 2024-05-28

**Authors:** Camelia Alexandra Coadă, Mihai Lupu, Iulia Florea, Stella Di Constanzo, Sara Coluccelli, Ioan Şimon

**Affiliations:** 1 Faculty of Medicine, “Iuliu Haţieganu” University of Medicine and Pharmacy Cluj-Napoca, 400012 Cluj-Napoca, Romania; coada_camelia_alexandra@elearn.umfcluj.ro (C.A.C.); iulia.florea@elearn.umfcluj.ro (I.F.); 2 Morpho-Functional Sciences Department, “Iuliu Haţieganu” University of Medicine and Pharmacy Cluj-Napoca, 400012 Cluj-Napoca, Romania; 3 Division of Oncologic Gynecology, IRCCS Azienda Ospedaliero-Universitaria di Bologna, 40138 Bologna, Italy; stella.dicostanzo@unibo.it; 4 Solid Tumor Molecular Pathology Laboratory, IRCCS Azienda Ospedaliero-Universitaria di Bologna, 40138 Bologna, Italy; sara.coluccelli@aosp.bo.it; 5 Department of Surgery, Faculty of Medicine, “Iuliu Haţieganu” University of Medicine and Pharmacy Cluj-Napoca, 400012 Cluj-Napoca, Romania; ioan.simon@umfcluj.ro

**Keywords:** stroke, cerebrovascular disease, genetic variant, PIA2 polymorphism, platelet glycoprotein

## Abstract

Cardiovascular diseases are the main cause of death in the world, with ischemic heart disease (i.e., myocardial infarction) and cerebrovascular disease (i.e., stroke) taking the highest toll. Advances in diagnosis and treatment have led to a significant alleviation of ischemic complications, specifically in the realm of pharmacotherapy and interventional devices, while pharmacogenomics has yet to be fully leveraged to improve the burden of disease. Atherothrombotic events might occur earlier or respond worse to treatment in patients with genetic variants of GP IIb/IIIa. Therefore, we aimed to quantitate the involvement of the PlA2 variant in the risk of cerebral stroke events. A systematic search and meta-analysis were performed by pooling the risks of individual studies. A total of 31 studies comprising 5985 stroke patients and 7886 controls were analyzed. A meta-analysis of four studies on hemorrhagic stroke patients showed no association with the PIA2 rs5918(C) polymorphism in both fixed-effect (OR = 0.90 95%CI [0.71; 1.14]; *p* = 0.398) and random-effect models (OR = 0.86 95%CI [0.62; 1.20]; *p*-value = 0.386). The power of this analysis was below <30%, indicating a limited ability to detect a true effect. An analysis of the 28 studies on ischemic stroke revealed a significant association with the PIA2 rs5918(C) allele in both fixed-effect (OR = 1.16 95%CI [1.06; 1.27]; *p* = 0.001) and random-effect models (OR = 1.20 95%CI [1.04; 1.38]; *p*-value = 0.012), with a power of >80%. The PIA2 allele was associated with an increased risk of ischemic stroke. No association was found with hemorrhagic stroke, most likely due to the small number of available studies, which resulted in a lack of power.

## 1. Introduction

Ischemic disease is a leading cause of death worldwide, with the heart and brain being the predilect targets of atherosclerosis [1]. While mortality is higher in the case of cardiac events [1], brain infarction leads to a more prominent burden on quality of life, as neurological cell death affects functional independence and multiple aspects of life, more so than cardiac failure [2].

Antithrombotic treatment is the mainstay therapy in the secondary prevention of is-chemic conditions, whereas interventional procedures lead to a decrease in the complications of acute events. Furthermore, advances in genome sequencing and the advent of personalized pharmacogenomics have shown discrepancies in treatment response in patients with different rheologic phenotypes, especially in the case of genes involved in hemostasis [3]. Because arterial clots tend to be platelet-rich (as opposed to venous’ clotting factor preponderance), clinical research has focused on primary hemostasis as a means to prevent arterial occlusions [3].

In the setting of endothelial disruption (atherosclerotic or otherwise) and shear stress, the hemostatic process is initiated by platelet adhesion and mediated by the von Willebrand factor (vWF) and glycoprotein (GP) Ib/Ia, which, in turn, leads to platelet aggregation and white thrombus formation, facilitated by GP IIb/IIIa. GP IIb/IIIa (also known as integrin αIIbβ3) is a platelet fibrinogen receptor with the form of a heterodimer (IIb and IIIa) that is assembled and stored in alpha granules inside platelets. It provides a key point between primary hemostasis and the coagulation cascade, as proven by its implication in both hemorrhagic and thrombotic events [4,5,6,7]. While patients with Glanzmann thrombasthenia exhibit higher rates of bleeding, single-nucleotide polymorphisms (SNP) of the IIIa subunit have been associated with an increased rate of platelet activation, earlier debut of atherosclerotic disease [4], and resistance to commonly used antiplatelet drugs (aspirin and clopidogrel) [8].

The most frequent SNP is rs5918 PlA1/A2 of the IIIa moiety and causes the replacement of leucine (PlA1) with proline (PlA2 variant), which has been evaluated in various studies regarding cardiac and brain ischemia, as well as other diseases [4,5,6,7]. Both ischemic and hemorrhagic stroke might be affected by GP IIb/IIIa variants, particularly in young patients, where the risk was evaluated to be higher in Caucasian women <45 years old, without reaching clear-cut statistical significance [9]. A decade-old meta-analysis appreciated that PlA2 homozygosity is positively associated with ischemic stroke, but not with hemorrhagic stroke [10]. Of note, all variants were observed to be clinically significant, mainly when associated with conventional cardiovascular risk factors such as diabetes, hypertension, or elevated homocysteine levels. This, together with the wide usage of GP IIb/IIIa inhibitors in cardiovascular disease [11], highlights the importance of a thorough investigation of the potential risks of its portents.

Over the years, the field of medical genetic testing has seen exponential growth as a result of ever-expanding knowledge, as well as increasingly affordable costs. Currently, there is a market for direct-to-consumer genetic panels, which are available to patients without the need for a medical prescription [12,13]. While this has exciting potential by enabling efficient screening for well-known cancer-associated germline mutations such as *BRCA1/2* mutations [14], panels aimed at multigenic and multifactorial traits may lack a robust foundation in studies, and consequently, their clinical utility raises concerns. Moreover, many such panels investigate polymorphisms rather than mutations, and are often based on small studies limited to specific populations without specifying their potential rarity in other populations [15].

Currently, the rs5918 PlA1/A2 SNP is included in various genetic testing panels dedicated to the exploration of cardiovascular risks. Therefore, the aim of this study was to conduct a meta-analysis of existing research to establish both the risk and magnitude of impact on the probability of developing a cerebral stroke. Moreover, we aimed to investigate whether the SNP is associated with a specific type of stroke.

## 2. Materials and Methods

### 2.1. Search Strategy and Article Identification

Public research databases were systematically queried for articles studying the relationship between the *GPIIIa* rs5918 and the risk of stroke. No limitations were imposed as to the type of stroke or the source of the population analyzed. The databases searched were PubMed, Scopus, Web of Science, and Cochrane Library. The GeneCards Human Gene Database [16] was used to identify all *GPIIIa* gene IDs’ synonyms. Synonyms of rs5918 were identified through Google Scholar search and SNP databases (dbSNP and SNPedia). Advanced search strategies were employed by combining relevant key terms: “stroke”, “cerebrovascular”, “brain”, “infarction”, “ischemia”, “hemorrhagic”, “polymorphism”, “BDPLT2”, “integrin beta3”, “ITGB3”, “CD61”, “GPIIIa”, “GP3A”, “BDPLT2“, “GT2”, “BDPLT16”, “BDPLT24”, “Human Platelet Antigen 1“, “HPA-1”, “rs5918”, “PIA1/A2”, “SNP”, “genotype”, “allele”, “mutation”, and “variant”. Given that not all authors followed the HGVS [17] recommendations to use the SNP annotation on the primary protein sequence, further searches were conducted by including both the L33P (mature protein) and canonical L59P (primary protein). No language restrictions were in place during this search phase. The search results were exported and then loaded in Rayyan [18] for duplicate removal and article screening and selection.

References from the identified relevant articles were also considered and manually searched for additional data. The last updated research was conducted on the 5 April 2024.

### 2.2. Article Selection and Inclusion

The articles were screened according to the Preferred Reporting Items for Systematic Reviews and Meta-Analyses (PRISMA) guidelines and included if they met specific criteria. Eligible studies were those that analyzed the presence of the rs5918 SNP in patients diagnosed with stroke, had a case–control design and reported either the genotypes and/or allele frequencies in both cases and controls, or provided the computed odds ratio (OR) for the stroke group. Papers were excluded if they investigated outcomes other than stroke such as different cardiovascular pathologies, were of the wrong study type (case reports, conference abstracts, letters, or opinions), and were written in languages other than English. If a study analyzed multiple outcomes including stroke, only the analysis considering the relationship between rs5918 and stroke was considered for inclusion in the meta-analysis. Additionally, when multiple control populations were present within a study, the one that was the most similar (i.e., similar background risk factors) to the stroke group was considered. After the initial screening, the full texts of the identified articles were read to select the manuscripts for final inclusion in the meta-analysis.

In the case of studies showing the presence of overlap between patients, either the most recent publication was considered or the one comprising a larger number of patients.

### 2.3. Data Extraction

Relevant data were extracted from each article and stored in a database. Information regarding the number of cases and controls, country of origin, ethnic background, age, sex, distribution of genotype, and allele frequencies between groups and ORs (odds ratios) (if available) was collected.

### 2.4. Quality Assessment

A quality assessment of the included studies was conducted using the Newcastle–Ottawa Scale (NOS). Two authors independently reviewed the articles and assigned points to each item according to the NOS manual, for a maximum of 9 points. Discrepancies were discussed with a third author when necessary. The studies were considered to be of a high quality—“good”—if their cumulative score was above 6, “fair” if the score was between 4 and 6, and “poor” if the score was between 0 and 3.

### 2.5. Statistical Analysis

Analysis was conducted in R version 4.3.2 (31 March 2023 ucrt)—”Eye Holes” (R Foundation, Vienna, Austria) [19]. Meta-analysis was conducted using the meta package [20], and Forest plots were used to present the results. The association between the presence of the rs5918 polymorphism and the risk of stroke was assessed by pooling the ORs and their corresponding 95% confidence intervals (CI). Both the fixed-effects and random-effects models were used to compute the pooled ORs. The heterogeneity of the included studies was evaluated using Tau^2^ for between-study variance and Higgin’s and Thompson’s *I*^2^ for the percentage of variability in the effect sizes not due to sampling error [21,22]. The Restricted Maximum-Likelihood estimator was used to calculate Tau^2^.

Publication bias was assessed using Egger’s regression and visualized using Funnel plots [23]. A power-enhanced sunset Funnel plot was graphed to examine the power of individual studies [24]. The overall power of the pooled results was determined using the dmetar R package [25].

## 3. Results

**Figure 1:**
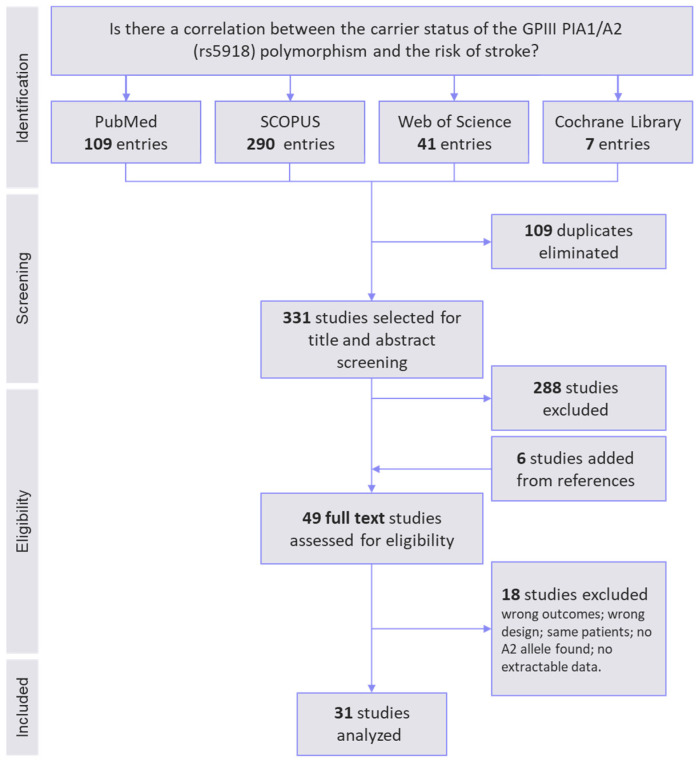
PRISMA flowchart illustrating the workflow for study identification, screening, eligibility evaluation, and inclusion in the meta-analysis. A total of 31 studies met the inclusion criteria and were pooled in the analysis.

The process and results of the identification, screening, and selection of the articles are presented in the PRISMA flowchart (Figure 1). Out of the 440 potentially relevant studies identified, 109 were duplicates and were eliminated. After title and abstract screening, 49 studies were selected for full text reading. Next, 17 studies were further discarded due to having a wrong outcome or design, overlapped patients, or no usable data. The study of Chen et al. [26] was eliminated from the analysis because they found no patient or control carriers of the SNP of interest. Finally, 31 studies were included in the meta-analysis, for a total number of 5985 patients diagnosed with stroke and a total of 7886 controls (Table 1).

### 3.1. Quality Assessment of the Included Studies

The NOS tool for case–control studies was applied to all 31 studies selected for the meta-analysis. The criteria with the highest points across studies were those in the “Selection” section, suggesting strength in the methodology of participant selection. Conversely, the area with the lowest points was that of “Comparability”. Nevertheless, the overall scores indicated that the studies were of a good quality. The assigned points for each item are reported in Supplementary Table S1.

### 3.2. Publication Bias and Power Estimation

**Figure 2:**
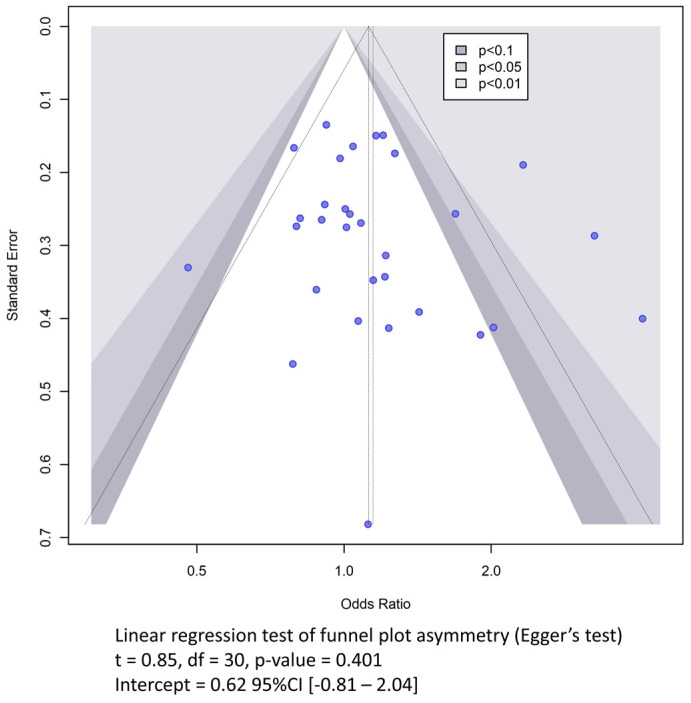
Funnel plot showing the publication bias. The vertical dashed line represents the average effect size while the diagonal dashed lines represent the idealized funnel shape that the studies are expected to follow. CI: confidence interval; df: degrees of freedom; t: t-statistic.

Publication bias was inspected through the construction of a funnel plot (Figure 2). There was a slight asymmetrical pattern given by four studies with high effect sizes, namely Saidi et al. [29], Roldan et al. [47], Derle et al. [32], and Iniesta et al. [35] (Supplementary Figure S1). However, testing for the presence of publication bias using Eggers’ test did not indicate a significant funnel plot asymmetry (t = 0.85, df = 30, *p*-value = 0.401). An examination of the power-enhanced funnel plot revealed that most of the published studies had a low power (Supplementary Figure S2). The overall power of this meta-analysis was calculated to be 95.59% for a fixed-effect model and 85% when assuming a random-effects model. When considering the meta-analysis conducted on studies with hemorrhagic stroke, the power was 20.14% for a fixed-effect model and 23.65% for a random-effects model. The meta-analysis of the ischemic stroke studies showed a power of 94.11% for the fixed-effect model and 82.15% for the random-effect model.

### 3.3. Carrier Status of the PIA2 rs5918(C) Allele and Stroke Risk

The selected studies comprised both hemorrhagic and ischemic stroke subtypes. Most of them focused on ischemic strokes (n = 28), while only four studies analyzed hemorrhagic strokes. Among these, one study included patients from both subtypes of stroke [9].

**Figure 3:**
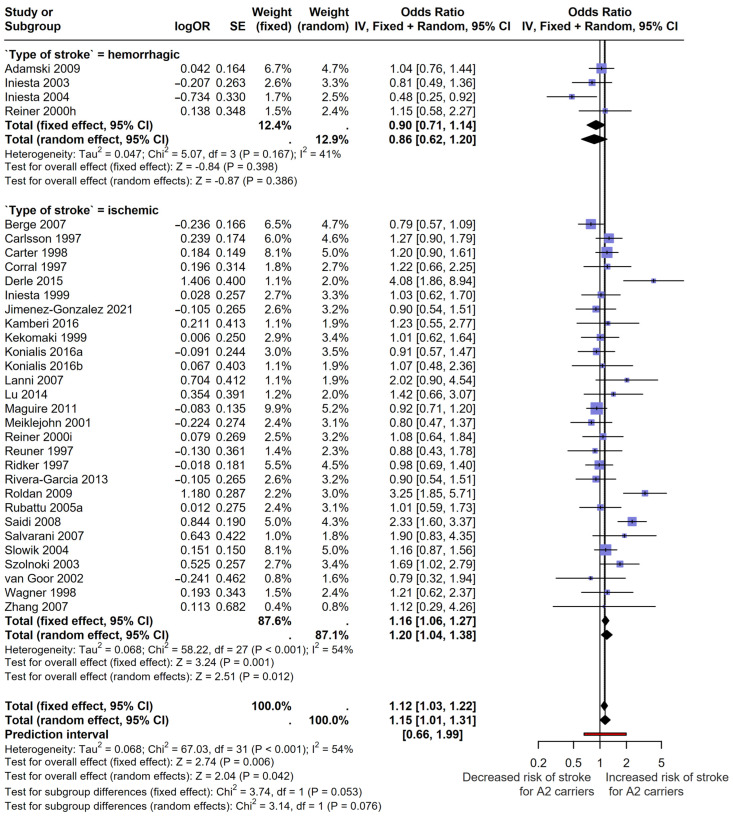
Forest plot showing the OR of individual studies [9,27,28,29,30,31,32,33,34,35,36,37,38,39,40,41,42,43,44,45,46,47,48,49,50,51,52,53,54] and the pooled analysis divided by type of stroke and overall effects. Both fixed effect and random effect models were used. OR: odds ratio; CI: confidence interval; SE: standard error.

A meta-analysis of the four studies [9,27,34,35] including hemorrhagic stroke patients showed no association with the carrier status of the PIA2 rs5918(C) polymorphism in both fixed-effect (OR = 0.90 95%CI [0.71; 1.14]; *p* = 0.398) and random-effect models (OR = 0.86 95%CI [0.62; 1.20]; *p*-value = 0.386). The *I*^2^ index showed a moderate but insignificant heterogeneity of the studies (*I*^2^ = 41% [0%; 80%]), Tau^2^ = 0.05 [0.00; 1.80]. Cochran’s Q was 5.06 with a *p* = 0.167. The prediction interval was wide [0.26; 2.83] (Figure 3; Supplementary Figure S3).

An analysis of the 28 studies [9,28,29,30,31,32,33,36,37,38,39,40,41,42,43,44,45,46,47,48,49,50,51,52,53,54] focusing on ischemic stroke revealed a significant association with the carrier status of the PIA2 rs5918(C) allele in both the fixed-effect (OR = 1.16 95%CI [1.06; 1.27]; *p* = 0.001) and random-effect models (OR = 1.20 95%CI [1.04; 1.38]; *p*-value = 0.012). The *I^2^* index showed a moderate/substantial percentage of effect sizes variation due to heterogeneity (*I*^2^ = 54% [29%, 70%]) and Tau^2^ = 0.07 [0.01, 0.20]. Cochran’s Q was 58.22 with a significant *p*-value < 0.001. The prediction interval was also wide [0.68; 2.09] (Figure 3; Supplementary Figure S4).

A total pooled analysis showed that carriers of the PIA2 rs5918(C) allele had a higher risk of stroke with an OR of 1.12 95%CI [1.03; 1.22], *p*-value = 0.006, when considering a fixed-effect model. In the random-effect model, the pooled OR was 1.15 95%CI [1.01; 1.31], *p*-value = 0.042. The heterogeneity index showed an *I*^2^ = 54% [31%; 69%], corresponding to a moderate/substantial heterogeneity and inconsistency across studies. Tau^2^ was 0.068 with its CI [0.02, 0.20], suggesting the presence of between-study heterogeneity and Q = 67.03 with a *p*-value < 0.001. Moreover, the prediction interval of the studies fell in the range of [0.66; 1.99], suggesting a high dispersion of the effects seen across individual studies (Figure 3).

A separate analysis was conducted on the studies reporting the ethnicity of the included subjects. A total of 16 studies included Caucasian subjects and two studies included Asian subjects. The meta-analysis of the 16 studies revealed an OR of 1.17 95%CI [1.02, 1.35] for both fixed and random effects; *p*-value = 0.026 (Supplementary Figures S5 and S6).

## 4. Discussion

Stroke represents a major health problem, as it ranks among the topmost contributors to global mortality and long-term disability [55,56]. The underlying pathophysiology of stroke encompasses multiple risk factors, both genetic as well as environmental factors [57]. Amid the non-genetic modifiable risk factors, the most well-known are heart disease, blood hypertension, sedentary lifestyle, smoking and alcohol consumption, dyslipidemia, and obesity [58,59]. These factors interact between themselves as well as with genetic predisposition factors in a complex manner, collectively contributing to its onset. Among the explored genetic risk factors, the PIA2 allele of GPIIIa has gained considerable attention, so much so that it is part of various genetic panels for cardiovascular risk assessment. We conducted a meta-analysis of the available studies to assess the relationship of this SNP with the risk of developing a stroke and found a small but significant association with the ischemic type, but not with the hemorrhagic type.

One important aspect that stands out from the pooling of the individual studies is that the effect sizes varied substantially across them. The implications of this variation need to be considered and discussed. Indeed, with a high number of studies, heterogeneity is inevitable [21]. However, the high heterogeneity here could denote that the mean effect may not be representative for all populations. This was further highlighted by the large width of the prediction interval which included 1. The wide dispersion of the PIA2 effects suggests that, at one extreme, the studies point out a trivial or even nil risk of stroke, while the other extreme points out an elevated risk of stroke. Moreover, most studies were conducted on Caucasians, further limiting the generalizability of these results.

### 4.1. The PIA2 Allele in the Context of Different Populations

It is worth discussing that allele frequency distribution among different populations is highly diverse, thus, studies tackling this topic should assess their required sample size in a population-dependent manner [60,61]. None of the studies included in this meta-analysis conducted an a priori sample size calculation, making most of the studies underpowered. This was confirmed by the power-enhanced funnel plot showing that the studies had a power less than 40%.

rs5918 is a relatively uncommon SNP present with different frequencies among populations. The Genome Aggregation Database (gnomAD) [62], a platform encompassing data from more than 800,000 exomes and genomes from various human sequencing studies, revealed that the minor allele frequencies (MAFs) of the rs5918 SNP are highest in Europeans, with MAFs of around 0.15, while the number significantly drops for East Asian populations, who report a MAF of 0.004. This explains the inexistence of PIA2 carriers in the study of Chen et al. [26], who conducted their research on cases from Taiwan, where this SNP is rare. Therefore, their study would have required an infeasibly large population to test the hypothesis. Another study, that of Zhang et al. [54], included 285 patients from China, where the MAF was reported to be around 0.09, and, in fact, had the lowest power of all studies included in our meta-analysis.

### 4.2. Clinical Relevance of the Results in the Context of Direct-to-Consumer Genetic Testing Panels

The issue of underpowered studies is widely spread, even among Cochrane reviews, impacting the precision and heterogeneity of results. Moreover, many meta-analyses themselves do not estimate their power [63]. The present meta-analysis conducted on the 28 studies with ischemic stroke patients had an adequate power of more than 80%. The pooled ORs for ischemic stroke studies were 1.16 and 1.20 for fixed- and random-effect models, respectively. This suggests that the impact, albeit statistically significant, may not be sufficiently large to offer practical clinical utility, and that testing for this SNP for stroke risk assessment may be futile in certain cases [64]. On the other hand, with only four studies available for the meta-analysis of hemorrhagic strokes, the statistical power was slightly above 20%, considerably lower than the recommended threshold. This discrepancy in the available literature is not surprising, since the vast majority of strokes are of an ischemic nature [1]. Nevertheless, the relationship between PIA2 and hemorrhagic stroke seemed to have an opposite trend. While these results need to be taken cautiously due to the underpowered meta-analysis, the pooled ORs were 0.86 and 0.90. Given that the PIA2 SNP is associated with an increase in platelet aggregability [65], it is not surprising that carriers may have some level of protection from hemorrhagic events.

Overall, one could argue that a genetic variant with such a high variability and such negligible impact should have limited clinical relevance, at least when interpreted alone. Indeed, the presence of several genetic variants with minor impacts alone could result in a significant cumulative impact, should they be present simultaneously in a patient [66]. However, to the best of our knowledge, many current genetic testing panels for multifactorial diseases lack compelling evidence for the genetic variants they include. Moreover, the reports accompanying the results are often scarce, without offering further genetic counseling [12,67]. Nowadays, direct-to-consumer genetic testing allows patients to order such tests without specific medical indication, exposing them to unintended information and to false-positive or -negative results [13,68]. The discovery of a health risk, albeit sometimes with low probabilities, can lead to psychological distress, as many patients do not fully grasp the notion that a risk does not imply that they will actually develop the respective disease [13,69]. Furthermore, the rapid growth and high availability of these panels has long surpassed the training of many medical doctors, limiting their capacity to accurately interpret their patients’ results [12,13,67]. Therefore, well-conducted systematic analyses such as the one presented here are much needed to fill in this knowledge gap.

### 4.3. Study Limitations and Future Directions

A relevant limitation of the analyzed studies is the heterogeneity of cases, and most importantly controls, which were taken both from the general population as well as from patients matched for comorbidities known to influence the risk of stroke, such as atrial fibrillation, body mass index, age, and other factors. This might also have contributed to the high variability and heterogeneity of the results. Despite this issue, strong predictors, whether genetic or not, should have a more pronounced association that is independent of other comorbidities.

Despite advancements in and the widespread availability of genetic testing, the evidence regarding the importance of the PIA1/A2 polymorphism in stroke risk remains modest. While a positive association with the risk of ischemic stroke was found, no progress in understanding its role in hemorrhagic stroke has been recorded. This issue was due to an insufficient number of adequately powered studies on this topic. Further research is needed to elucidate the association between PIA2 and this type of stroke.

## 5. Conclusions

The carrier status for the PIA2 polymorphism was significantly associated with a marginally elevated risk of ischemic stroke. No significant association could be found with hemorrhagic stroke, due to the small number of studies analyzing such patients. Care must be taken when evaluating the risk of stroke through genetic testing, due to its multifactorial nature.

## Figures and Tables

**Table 1:** Description of the studies included in the meta-analysis. N: number.

Author, Year	Country/Race	Type of stroke	No. of Stroke Cases/Controls	Selection Criteria of Stroke Cases/Controls
Adamski et al., 2009 [27]	Poland Caucasian	Hemorrhagic	288/457	White patients with aneurysmal SAH recruited between 2001 and 2007 at the Stroke Unit and the Department of Neurosurgery, Jagiellonian University, Krakow, Poland. Controls matched by gender, race, and age (±10 years) without any history of cerebrovascular disease.
Berge et al., 2007 [28]	Norway	Ischemic	363/482	Patients with acute ischemic stroke and atrial fibrillation. Unselected healthy blood donors recruited from the Blood Bank.
Carlsson et al., 1997 [29]	Germany	Ischemic	218/486	Patients from 1995–1996, admitted to the stroke service of the Department of Neurology. Subarachnoid hemorrhage or patients with a stroke due to vasculitis were excluded. Control group were neurological inpatients without signs for vascular pathology and healthy blood donors.
Carter et al., 1998 [30]	England Caucasian	Ischemic	505/402	Clinical diagnosis of acute stroke. Control subjects were healthy white Europeans free from vascular disease.
Corral et al., 1997 [31]	Spain	Ischemic	103/103	Unselected patients with cerebrovascular disease with age-, race-, and sex-matched controls with no history of cerebrovascular disease.
Derle et al., 2015 [32]	Türkiye	ischemic	42/133	Patients with acute ischemic stroke or TIA. Controls were patients at a high risk for vascular events but with a negative history.
Iniesta et al., 1999 [33]	Spain	Ischemic	124/236	Patients diagnosed with ischemic attack, minor stroke, and cerebral infarction. Controls were individuals with no documented history of migraine, venous, and/or arterial thrombosis and sex- and age-matched individuals with the same risk factors as the patients with ischemic CVD and migraine.
Iniesta et al., 2003 [34]	Spain Caucasian	Hemorrhagic	141/141	Adult patients with a first episode of PIH. Controls were matched by age, sex, race, and selected risk factors for PIH (smoking history, blood pressure, and alcohol consumption), without a documented history of vascular disease.
Iniesta et al., 2004 [35]	Spain Caucasian	Hemorrhagic	103/103	Patients diagnosed with aneurysmal and spontaneous nonaneurysmal SAH. Controls were subjects without a documented history of vascular disease nor a personal history of thromboembolic or hemorrhagic disease and were not undergoing antithrombotic therapy.
Jimenez-Gonzalez et al., 2021 [36]	Mexico	Ischemic	200/200	CT-confirmed diagnosis of ischemic CVD without risk factors for cardioembolic CVD. Controls were healthy potential blood bank donors with no relevant history of CVD and normal biochemical parameters.
Kamberi et al., 2016 [37]	Macedonia Caucasian	Ischemic	39/102	Consecutive patients admitted to the Neurology Department with confirmed diagnosis acute first IS, (age: 18–90 years). Controls were subjects free from CVD.
Kekomaki et al., 1999 [38]	Finland Caucasian	Ischemic	234/200	Survivors of IS or transient ischemic attack. Controls had no signs of CVD and also lived in North Karelia.
Konialis et al., 2016a [39]	Greece Caucasian	Ischemic	166/159	Adult patients newly diagnosed with IS. Control group: adult participants entering the hospital for a confirmed reason unrelated to cerebro- or cardiovascular disease.
Konialis et al., 2016b [39]	Greece Caucasian	Ischemic	34/165	Adult patients newly diagnosed with IS and CHD.
Lanni et al., 2007 [40]	Italy Caucasian	Ischemic	28/180	Hypertensive individuals with IS. Control group were hypertensive individuals without ischemic stroke.
Lu et al., 2014 [41]	China Asian	Ischemic	350/300	Included IS patients (males and females, mean age, 55 years), some with a smoking history and an alcohol consumption history. Controls were healthy people who received physical examination in hospital during the same time period.
Maguire et al., 2011 [42]	Australia Caucasian (94.3%) and non-Caucasian	Ischemic	609/627	Patients with IS, as defined by World Health Organization clinical criteria. Controls were random healthy community men and women (age 55–85) from the New South Wales Electoral Roll between 2004 and 2007.
Meiklejohn et al., 2001 [43]	Scotland Caucasian	Ischemic	150/150	Patients with a cerebrovascular accident without a history of atrial fibrillation, valvular heart disease, or connective tissue disease. Controls were patients born in same year as the subjects with no history of stroke, TIA, peripheral vascular, or ischemic heart disease.
Reiner et al., 2000 [9]	USA Caucasian	Ischemic and hemorrhagic	78 (42 + 36)/346	Caucasian women with nonfatal stroke with brain imaging signs from the Baltimore-Washington Cooperative Young Stroke Study.
Reuner et al., 1997 [44]	Germany	Ischemic	79/79	Patients were male and female with stroke or with TIA. Controls were age- and sex-matched healthy subjects without history of arterial vascular disease.
Ridker et al., 1997 [45]	USA	Ischemic	209/704	Subjects from the Physicians’ Health Study randomized, double-blind, placebo-controlled trial. Men aged 40–84, with no history of myocardial infarction, stroke, TIA, or cancer who developed IS. Control group were subjects without signs of CVD.
Rivera-Garcia et al., 2013 [46]	Mexico/ Mexican	Ischemic	200/200	Consecutive unrelated patients <45 years old, with diagnosis of IS. The control group was constituted by individuals without history of CVD.
Roldan et al., 2009 [47]	Spain	Ischemic	96/119	Patients with atrial fibrillation with or without IS. Cases with recent venous thromboembolism, myocardial infarction, acute coronary syndrome, infection or inflammatory disease, surgery, malignancy, renal/liver impairment, and with hormone replacement therapy or oral anticoagulation were excluded.
Rubattu et al., 2005 [48]	Italy	Ischemic	115/180	First-ever IS patient. Patients with TIA were excluded. Controls were age-matched healthy unrelated subjects recruited from the same medical center, among a population of blood donors. They were free from any pharmacological treatment.
Saidi et al., 2008 [29]	Tunisia Arabs	Ischemic	329/444	Tunisian hospitalized patients with first IS from the Neurology service of CHU Sahloul, Sousse, Tunisia. Controls were unrelated healthy individuals from the same geographical area as the patients.
Slowik et al., 2004 [49]	Poland Caucasian	Ischemic	377/572	Unrelated patients with IS. Controls were unrelated individuals, free of clinically detectable CVD and without any stroke history.
Salvarani et al., 2007 [50]	Italy Caucasian	Ischemic	31/109	Patients with biopsy-proven giant cell arteritis who were residents of Reggio Emilia, Italy, with cranial ischemic complications. Controls were population-based healthy subjects from the same geographic area.
Szolnoki et al., 2003 [51]	Hungary Caucasian	Ischemic	545/158	Consecutive Caucasian Hungarian patients with a first acute IS. Controls were random neurological patients without stroke and with normal MRI.
van Goor et al., 2002 [52]	Netherlands	Ischemic	45/60	Patients with TIA and ischemic stroke. Controls were healthy blood donors. Risk factors and clinical data were not assessed.
Wagner et al., 1998 [53]	USA Caucasian Black	Ischemic	65 (33 + 32)/ 123 (76 + 47)	Female patients from 15 to 44 years of age with a first cerebral infarction, identified by discharge surveillance at 59 regional hospitals and through direct referral by regional neurologists. Controls were women without a history of stroke, frequency-matched by age and geographic region of residence to the cases.
Zhang et al., 2007 [54]	China Asian	Ischemic	119/166	Patients with a diagnosis of IS. Cardioembolic strokes, hemorrhagic stroke, TIA, cerebral venous thrombosis, and hemorrhagic transformation of an infarct were excluded. Controls were age- and sex-matched apparently healthy subjects without a previous history of stroke, myocardial infarction and peripheral arterial disease.

SAH: subarachnoid hemorrhage; CVD: cardiovascular disease; IS: ischemic stroke; CHD: coronary heart disease; and TIA: transient ischemic attack.

## Data Availability

All data generated in this work is presented in the manuscript.

## Supplementary Materials

The following supporting information can be downloaded at: https://www.mdpi.com/article/10.3390/cimb46060321/s1. Table S1: Quality assessment of the studies included in the meta-analysis; Figure S1: Funnel plot with labels for the inspection of study bias showing the scatter of the studies effect sizes and the measure of their standard error; Figure S2: Sunset power enhanced funnel plot showing study-level statistical power; Figure S3: Forest plot for haemorrhagic stroke studies; Figure S4: Forest plot for ischemic stroke studies; Figure S5: Forest plot for stroke studies specifying the race: Caucasian; Figure S6: Forest plot for stroke studies specifying the race: Asian.
