# Supplementary material for: Association of Glycoprotein IIIa PlA1/A2 Polymorphism with Risk of Stroke: Updated Meta-Analysis
Source: Curr Issues Mol Biol. 2024 May 28;46(6):5364–78. doi: 10.3390/cimb46060321 (PMC11201703; doi:10.3390/cimb46060321)
Supplement: Supplementary file 1 [file cimb-46-00321-s001.zip › cimb-3021629-supplementary.pdf]

Supplementary Table S1. Quality assessment of the studies included in the meta-analysis.

Item	Selection				Comparability		Exposure			Total	Overall rating
**Author, year**	**1**	**2**	**3**	**4**			**1**	**2**	**3**	**(max 9)**	
Adamski et al. 2009 [27]	1	1	1	1	1	0	1	1	1	8	good
Berge et al. 2007 [28]	1	1	0	1	1	0	1	1	1	7	good
Carlsson et al. 1997 [29]	1	1	1	1	1	0	1	1	1	8	good
Carter et al. 1998 [30]	1	1	1	1	1	1	1	1	1	9	good
Corral et al. 1997 [31]	1	1	0	1	1	1	1	1	1	8	good
Derle et al. 2015 [32]	1	1	1	1	1	0	1	1	1	8	good
Iniesta et al. 1999 [33]	1	1	1	1	1	1	1	1	1	9	good
Iniesta et al. 2003 [34]	1	1	0	1	1	1	1	1	1	8	good
Iniesta et al. 2004 [35]	1	1	0	1	1	1	1	1	1	8	good
Jimenez-Gonzalez et al. 2021 [36]	1	1	1	1	1	0	1	1	1	8	good
Kamberi et al. 2016 [37]	1	1	1	1	1	1	1	1	1	9	good
Kekomaki et al. 1999 [38]	1	1	1	1	1	1	1	1	1	9	good
Konialis et al. 2016a [39]	1	1	1	1	1	1	1	1	1	9	good
Konialis et al. 2016b [39]	1	1	1	1	1	1	1	1	1	9	good
Lanni et al. 2007 [40]	1	1	0	1	1	1	1	1	1	8	good
Lu et al. 2014 [41]	1	1	1	1	1	0	1	1	1	8	good
Maguire et al. 2011 [42]	1	1	1	1	1	0	1	1	1	8	good
Meiklejohn et al. 2001 [43]	1	1	1	1	1	0	1	1	1	8	good
Reiner et al. 2000 [9]	1	1	1	1	1	1	1	1	1	9	good
Reuner et al. 1997 [44]	1	1	1	1	1	0	1	1	1	8	good
Ridker et al. 1997 [45]	1	1	1	1	1	0	1	1	1	8	good
Rivera-Garcia et al. 2013 [46]	1	1	1	1	1	1	1	1	1	9	good
Roldan et al. 2009 [47]	1	1	1	1	1	1	1	1	1	9	good
Rubattu et al. 2005 [48]	1	1	1	1	1	1	1	1	1	9	good
Saidi et al. 2008 [29]	1	1	1	1	1	0	1	1	1	8	good
Slowik et al. 2004 [49]	1	1	1	1	1	1	1	1	1	9	good
Salvarani et al. 2007 [50]	1	1	1	1	1	0	1	1	1	8	good
Szolnoki et al. 2003 [51]	1	1	1	1	1	1	1	1	1	9	good
van Goor et al. 2002 [52]	1	1	1	1	0	0	1	1	1	7	good
Wagner et al. 1998 [53]	1	1	1	1	1	1	1	1	1	9	good
Zhang et al. 2007 [54]	1	1	1	1	1	1	1	1	1	9	good

The overall rating of "good," "fair," or "poor" "quality was given to the studies as follows: good - 7 to 9, fair - 4 to 6, and poor - 0 to 3.

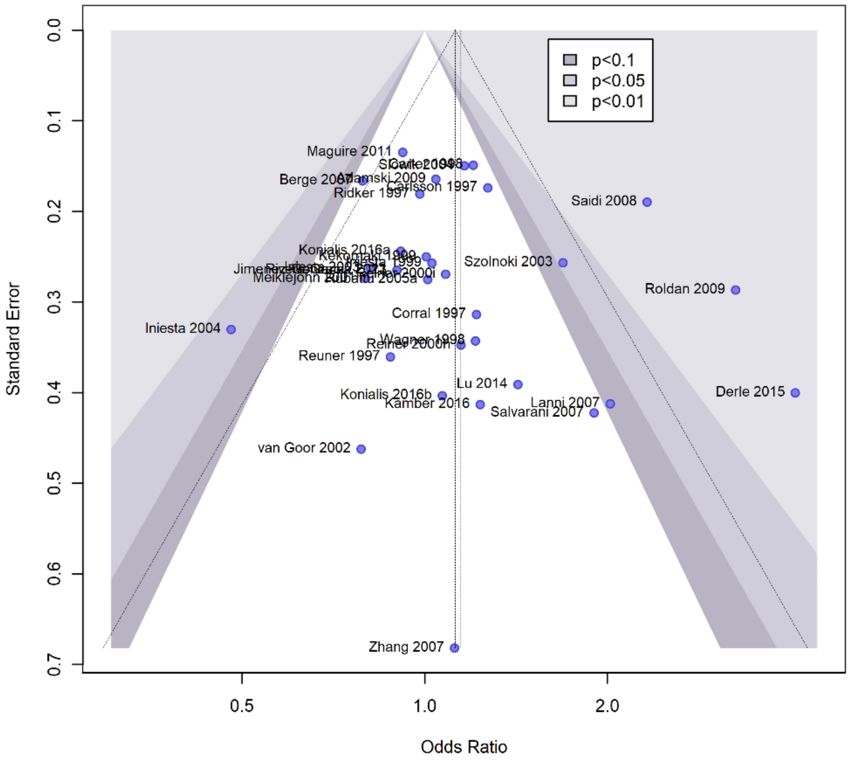

Supplementary figure S1. Funnel plot with labels for the inspection of study bias showing the scatter of the studies effect sizes and the measure of their standard error.

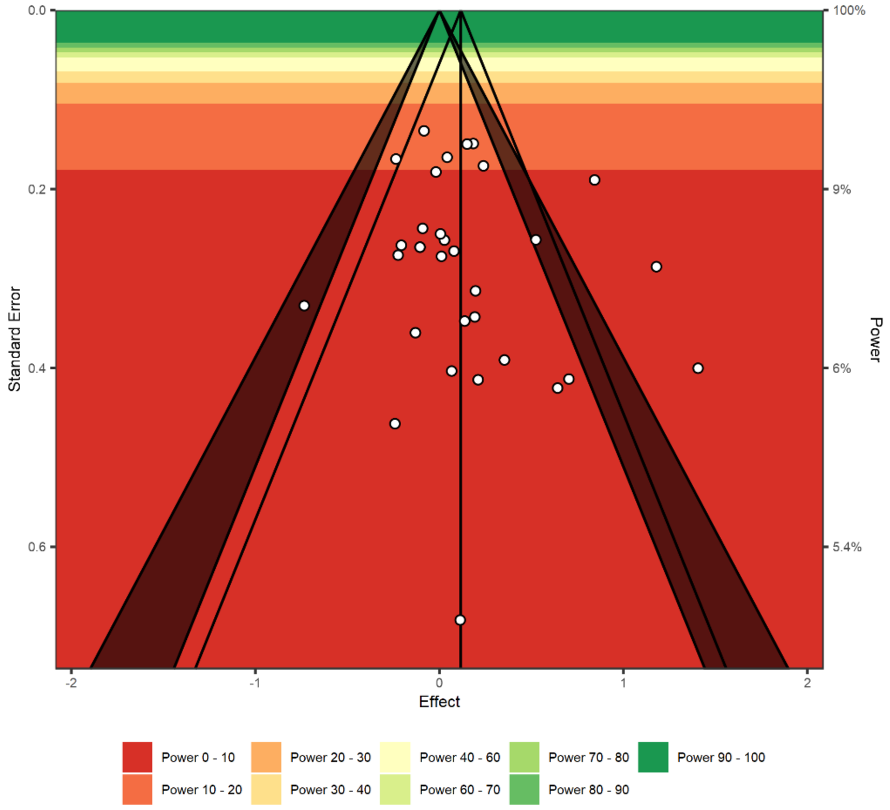

Supplementary figure S2. Sunset power enhanced funnel plot showing study-level statistical power.

Study	logOR	SE	Weight (fixed)	Weight (random)	Odds Ratio IV, Fixed + Random, 95% CI
Adamski 2009	0.042	0.164	53.7%	39.1%	1.04 [0.76, 1.44]
Iniesta 2003	-0.207	0.263	21.0%	25.0%	0.81 [0.49, 1.36]
Iniesta 2004	-0.734	0.330	13.3%	18.6%	0.48 [0.25, 0.92]
Reiner 2000h	0.138	0.348	12.0%	17.3%	1.15 [0.58, 2.27]
**Total (fixed effect, 95% CI)**			**100.0%**	**--**	**0.90 [0.71, 1.14]**
**Total (random effect, 95% CI)**			**--**	**100.0%**	**0.86 [0.62, 1.20]**
**Prediction interval**					**[0.26, 2.83]**

Heterogeneity: $Tau^2 = 0.047$; $Chi^2 = 5.07$, df = 3 (P = .167); $I^2 = 41\%$
Test for overall effect (fixed effect): Z = -0.84 (P = .398)
Test for overall effect (random effects): Z = -0.87 (P = .386)

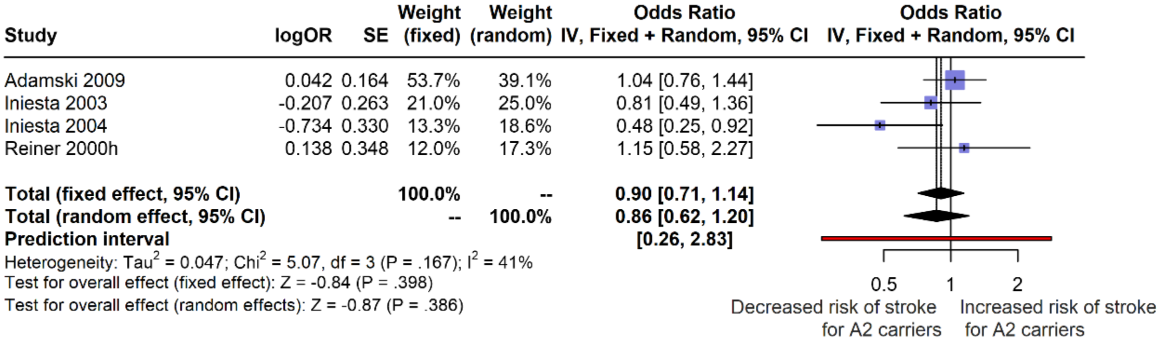

Supplementary figure S3. Forest plot for haemorrhagic stroke studies.

Study	logOR	SE	Weight (fixed)	Weight (random)	Odds Ratio IV, Fixed + Random, 95% CI
Berge 2007	-0.236	0.166	7.4%	5.4%	0.79 [0.57, 1.09]
Carlsson 1997	0.239	0.174	6.8%	5.2%	1.27 [0.90, 1.79]
Carter 1998	0.184	0.149	9.3%	5.7%	1.20 [0.90, 1.61]
Corral 1997	0.196	0.314	2.1%	3.1%	1.22 [0.66, 2.25]
Derle 2015	1.406	0.400	1.3%	2.3%	4.08 [1.86, 8.94]
Iniesta 1999	0.028	0.257	3.1%	3.8%	1.03 [0.62, 1.70]
Jimenez-Gonzalez 2021	-0.105	0.265	2.9%	3.7%	0.90 [0.54, 1.51]
Kamber 2016	0.211	0.413	1.2%	2.2%	1.23 [0.55, 2.77]
Kekomaki 1999	0.006	0.250	3.3%	3.9%	1.01 [0.62, 1.64]
Konialis 2016a	-0.091	0.244	3.5%	4.0%	0.91 [0.57, 1.47]
Konialis 2016b	0.067	0.403	1.3%	2.2%	1.07 [0.48, 2.36]
Lanni 2007	0.704	0.412	1.2%	2.2%	2.02 [0.90, 4.54]
Lu 2014	0.354	0.391	1.3%	2.3%	1.42 [0.66, 3.07]
Maguire 2011	-0.083	0.135	11.3%	6.0%	0.92 [0.71, 1.20]
Meiklejohn 2001	-0.224	0.274	2.7%	3.6%	0.80 [0.47, 1.37]
Reiner 2000i	0.079	0.269	2.8%	3.7%	1.08 [0.64, 1.84]
Reuner 1997	-0.130	0.361	1.6%	2.6%	0.88 [0.43, 1.78]
Ridker 1997	-0.018	0.181	6.3%	5.1%	0.98 [0.69, 1.40]
Rivera-Garcia 2013	-0.105	0.265	2.9%	3.7%	0.90 [0.54, 1.51]
Roldan 2009	1.180	0.287	2.5%	3.4%	3.25 [1.85, 5.71]
Rubattu 2005a	0.012	0.275	2.7%	3.6%	1.01 [0.59, 1.73]
Saidi 2008	0.844	0.190	5.7%	5.0%	2.33 [1.60, 3.37]
Salvarani 2007	0.643	0.422	1.2%	2.1%	1.90 [0.83, 4.35]
Slowik 2004	0.151	0.150	9.2%	5.7%	1.16 [0.87, 1.56]
Szolnoki 2003	0.525	0.257	3.1%	3.8%	1.69 [1.02, 2.79]
van Goor 2002	-0.241	0.462	1.0%	1.8%	0.79 [0.32, 1.94]
Wagner 1998	0.193	0.343	1.8%	2.8%	1.21 [0.62, 2.37]
Zhang 2007	0.113	0.682	0.4%	1.0%	1.12 [0.29, 4.26]
**Total (fixed effect, 95% CI)**			**100.0%**	**--**	**1.16 [1.06, 1.27]**
**Total (random effect, 95% CI)**			**--**	**100.0%**	**1.20 [1.04, 1.38]**
**Prediction interval**					**[0.69, 2.09]**

Heterogeneity: $Tau^2 = 0.068$; $Chi^2 = 58.22$, df = 27 (P < .001); $I^2 = 54\%$
Test for overall effect (fixed effect): Z = 3.24 (P = .001)
Test for overall effect (random effects): Z = 2.51 (P = .012)

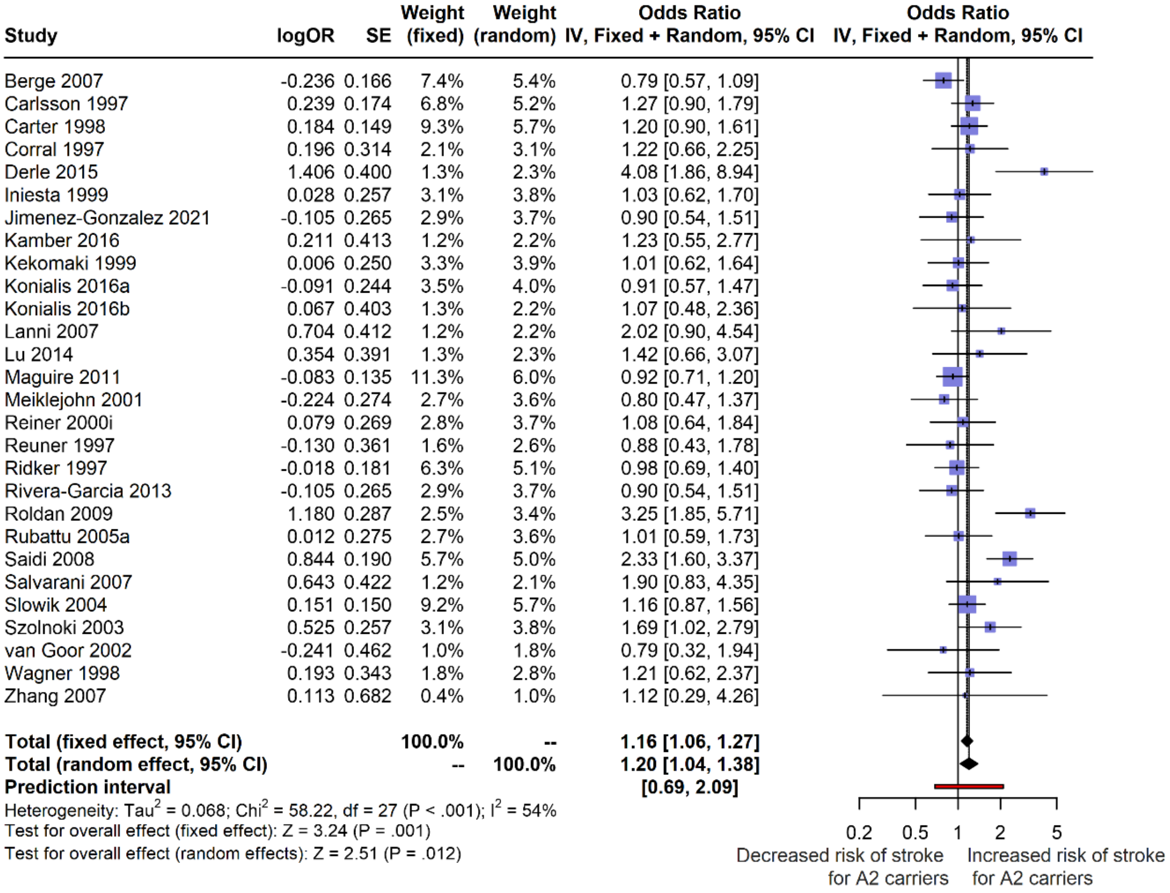

Supplementary figure S4. Forest plot for ischemic stroke studies.

Study or Subgroup	logOR	SE	Weight (fixed)	Weight (random)	Odds Ratio IV, Fixed + Random, 95% CI
**`Type of stroke` = hemorrhagic**					
Adamski 2009	0.042	0.164	14.1%	12.8%	1.04 [0.76, 1.44]
Iniesta 2003	-0.207	0.263	5.5%	6.1%	0.81 [0.49, 1.36]
Iniesta 2004	-0.734	0.330	3.5%	4.0%	0.48 [0.25, 0.92]
Reiner 2000h	0.138	0.348	3.2%	3.7%	1.15 [0.58, 2.27]
**Total (fixed effect, 95% CI)**			**26.3%**	**.**	**0.90 [0.71, 1.14]**
**Total (random effect, 95% CI)**			**.**	**26.6%**	**0.86 [0.62, 1.20]**
Heterogeneity: $Tau^2 = 0.047$; $Chi^2 = 5.07$, df = 3 (P = 0.167); $I^2 = 41\%$					
Test for overall effect (fixed effect): Z = -0.84 (P = 0.398)					
Test for overall effect (random effects): Z = -0.87 (P = 0.386)					
**`Type of stroke` = ischemic**					
Carter 1998	0.184	0.149	17.2%	14.7%	1.20 [0.90, 1.61]
Kamberi 2016	0.211	0.413	2.2%	2.7%	1.23 [0.55, 2.77]
Kekomaki 1999	0.006	0.250	6.1%	6.6%	1.01 [0.62, 1.64]
Konialis 2016a	-0.091	0.244	6.4%	6.9%	0.91 [0.57, 1.47]
Konialis 2016b	0.067	0.403	2.3%	2.8%	1.07 [0.48, 2.36]
Lanni 2007	0.704	0.412	2.2%	2.7%	2.02 [0.90, 4.54]
Meiklejohn 2001	-0.224	0.274	5.1%	5.6%	0.80 [0.47, 1.37]
Reiner 2000i	0.079	0.269	5.3%	5.8%	1.08 [0.64, 1.84]
Salvarani 2007	0.643	0.422	2.1%	2.6%	1.90 [0.83, 4.35]
Slowik 2004	0.151	0.150	17.0%	14.6%	1.16 [0.87, 1.56]
Szolnoki 2003	0.525	0.257	5.8%	6.3%	1.69 [1.02, 2.79]
Wagner 1998C	0.405	0.457	1.8%	2.2%	1.50 [0.60, 3.60]
**Total (fixed effect, 95% CI)**			**73.7%**	**.**	**1.17 [1.02, 1.35]**
**Total (random effect, 95% CI)**			**.**	**73.4%**	**1.17 [1.02, 1.35]**
Heterogeneity: $Tau^2 = 0$; $Chi^2 = 8.95$, df = 11 (P = 0.627); $I^2 = 0\%$					
Test for overall effect (fixed effect): Z = 2.23 (P = 0.026)					
Test for overall effect (random effects): Z = 2.23 (P = 0.026)					
**Total (fixed effect, 95% CI)**			**100.0%**	**.**	**1.10 [0.97, 1.24]**
**Total (random effect, 95% CI)**			**.**	**100.0%**	**1.09 [0.95, 1.25]**
**Prediction interval**					**[0.84, 1.43]**
Heterogeneity: $Tau^2 = 0.011$; $Chi^2 = 17.51$, df = 15 (P = 0.289); $I^2 = 14\%$					
Test for overall effect (fixed effect): Z = 1.48 (P = 0.138)					
Test for overall effect (random effects): Z = 1.27 (P = 0.204)					
Test for subgroup differences (fixed effect): $Chi^2 = 3.50$, df = 1 (P = 0.062)					
Test for subgroup differences (random effects): $Chi^2 = 2.78$, df = 1 (P = 0.096)					

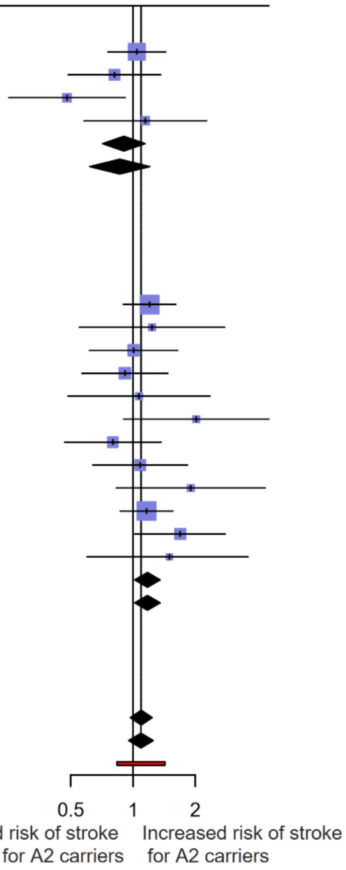

Supplementary figure S5. Forest plot for stroke studies specifying the race: Caucasian.

Study	logOR	SE	Weight (fixed)	Weight (random)	Odds Ratio IV, Fixed + Random, 95% CI
Lu 2014	0.354	0.391	75.2%	75.2%	1.42 [0.66, 3.07]
Zhang 2007	0.113	0.682	24.8%	24.8%	1.12 [0.29, 4.26]
**Total (fixed effect, 95% CI)**			**100.0%**	**.**	**1.34 [0.69, 2.61]**
**Total (random effect, 95% CI)**			**.**	**100.0%**	**1.34 [0.69, 2.61]**
Heterogeneity: $Tau^2 = 0$; $Chi^2 = 0.09$, df = 1 (P = 0.760); $I^2 = 0\%$					
Test for overall effect (fixed effect): Z = 0.87 (P = 0.386)					
Test for overall effect (random effects): Z = 0.87 (P = 0.386)					

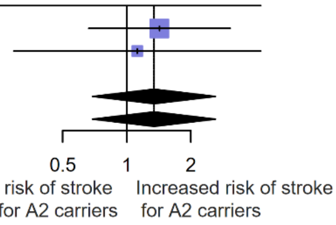

Supplementary figure S6. Forest plot for stroke studies specifying the race: Asian.
